# Information-Rich Multi-Functional OCT for Adult Zebrafish Intra- and Extracranial Imaging

**DOI:** 10.3390/bioengineering10070856

**Published:** 2023-07-19

**Authors:** Di Yang, Weike Wang, Zhuoqun Yuan, Yanmei Liang

**Affiliations:** Tianjin Key Laboratory of Micro-Scale Optical Information Science and Technology, Institute of Modern Optics, Nankai University, Tianjin 300350, China; 9820210118@nankai.edu.cn (D.Y.); wangweike@mail.nankai.edu.cn (W.W.); 1120210110@mail.nankai.edu.cn (Z.Y.)

**Keywords:** zebrafish, cranial imaging, optical coherence tomography (OCT), optical coherence tomography angiography (OCTA), polarization-sensitive optical coherence tomography (PS-OCT)

## Abstract

The zebrafish serves as a valuable animal model for both intra- and extracranial research, particularly in relation to the brain and skull. To effectively investigate the development and regeneration of adult zebrafish, a versatile in vivo imaging technique capable of showing both intra- and extracranial conditions is essential. In this paper, we utilized a high-resolution multi-functional optical coherence tomography (OCT) to obtain rich intra- and extracranial imaging outcomes of adult zebrafish, encompassing pigmentation distribution, tissue-specific information, cranial vascular imaging, and the monitoring of traumatic brain injury (TBI). Notably, it is the first that the channels through the zebrafish cranial suture, which may have a crucial function in maintaining the patency of the cranial sutures, have been observed. Rich imaging results demonstrated that a high-resolution multi-functional OCT system can provide a wealth of novel and interpretable biological information for intra- and extracranial studies of adult zebrafish.

## 1. Introduction

Due to its significant anatomical similarities and homology with humans, the zebrafish has emerged as a crucial animal model for both intra- and extracranial studies including the skull and brain [1,2,3]. Furthermore, due to the high regenerative capacity of the adult zebrafish’s brain and skull, this animal model holds great potential for elucidating human regeneration defects and advancing the field of regenerative medicine [4,5,6]. Moreover, because many mature organs and tissues can only be visualized in adult zebrafish [7,8], such as cranial sutures, there has been increasing interest in utilizing adult zebrafish to study intra- and extracranial development and regeneration [9,10].

Although the adult zebrafish has attracted significant interest in development and biomedical studies, in vivo imaging on adult zebrafish remains extremely challenging. Currently, fluorescence microscopy [11,12,13] is the main modality to conduct in vivo intra- and extracranial imaging, which offers cellular resolution and various molecular information. However, on the one hand, complex manipulations or transparent species are always needed since most species of adult zebrafish are opaque [11,12]. On the other hand, the spectral overlap between fluorescent labels restricts the number of biomarkers that can be used in the same individual [14]. Consequently, fluorescence microscopy faces a challenge in providing a comprehensive view of tissues or organs, as it is constrained by the availability of markers, the zebrafish species, and the imaging depth. Limited to this challenge, the understanding of adult zebrafish’s physiological structure is still incomplete, and new physiological structures are still being discovered, such as the newly described intracranial lymphatic vasculature [12]. Therefore, it is imperative to develop versatile imaging modalities to augment the comprehension of intra- and extracranial structures and development.

Optical coherence tomography (OCT) is a three-dimensional label-free imaging technology and has gained widespread attention in zebrafish research [15]. Its large field of view and high-resolution render OCT a promising tool for investigating intra- and extracranial physiological structures of zebrafish [16,17,18]. However, compared to fluorescence microscopy, conventional OCT lacks molecular specificity and typically necessitates additional functional information to differentiate various tissues. Polarization-sensitive optical coherence tomography (PS-OCT) and optical coherence tomography angiography (OCTA) serve as functional extensions of OCT, offering polarization and vascular contrasts, which can moderately improve the specific imaging ability of OCT. At present, PS-OCT has been initially applied to the imaging of the zebrafish brain. Our previous study investigated the polarization characteristics of the zebrafish brain and skull using PS-OCT [19]. Moreover, Antonia Lichtenegger et al. studied the polarization properties of zebrafish brain tumors [20]. While OCTA has yet to be employed for intra- or extracranial vascular imaging in adult zebrafish, our prior investigation has evinced its potential as a tool for imaging the vasculature of adult zebrafish skin [21]. Given the burgeoning interest in functional OCT-related research [15], the integration of polarization and vascular characteristics of tissues to generate comprehensible functional information has emerged as a pressing issue to be addressed.

In this study, a high-resolution multi-functional optical coherence tomography (OCT) was employed to perform intra- and extracranial imaging of adult zebrafish. Our imaging approach, which incorporated an organ segmentation method, enabled us to obtain polarization and vascular information on various organs. Through the analysis of multi-functional information, we achieved pigmentation distribution, tissue-specific information, cranial vascular imaging, and monitoring of traumatic brain injury (TBI). The imaging results revealed a range of physiological structures and pathophysiological processes. Notably, it is the first time the channels connecting intra- and extracranial vasculatures through cranial sutures have been observed which is a novel finding. It is anticipated that a high-resolution multi-functional OCT will offer diverse and innovative biological insights for zebrafish research, thereby enhancing the comprehension of intra- and extracranial development.

## 2. Materials and Methods

### 2.1. Multi-Functional OCT Imaging System with an Anesthesia System

The multi-functional OCT imaging system with an anesthesia system was described in detail in our previous study [19]. The schematic of the multi-functional OCT imaging system was described in Figure S1. The axial resolution of this imaging system is ~3.4 μm in air. The three-dimensional field of view is 3 × 3 × 2.3 mm (*x* × *y* × *z*). The optical power on the sample is ~4 mW. In this study, we set the transverse resolution to 8 μm and the spectrometer line rate to 25 kHz.

The schematic of the anesthesia system was described in Figure S2a. In a petri dish, a zebrafish was placed on the designed holder. The input end of the peristaltic pump is fixed to the bottom of the petri dish with waterproof tape. The zebrafish was intubated with the output end of the peristaltic pump, and the flow rate was set to ∼4 mL/min with the peristaltic pump. The photo of the anesthesia system is shown in Figure S2b.

### 2.2. Zebrafish Husbandry and Preparation

Zebrafish (*Danio rerio*) adults are kept according to standard laboratory conditions. Maintenance of adult zebrafish in the fish facilities is conducted at 28 °C, with a 14:10 (hour) light:dark cycle. The research was performed following the Guidelines for Care and Use of Laboratory Animals of Nankai University and was approved by the Laboratory Animal Ethics Committee of Nankai University.

For inducing a zebrafish traumatic brain injury, we used a similar method in our previous study [19]. Adult zebrafish were anesthetized in 0.02% tricaine solution. During anesthesia, a 30 G blood test needle was used to push through the skull for the injury. After being injured, the zebrafish was moved to the tank with fresh water until movement of the operculum is observed.

### 2.3. Image Acquisition and Processing

To conduct in vivo imaging, the zebrafish was anesthetized and positioned on a designed holder within a petri dish. A peristaltic pump with a slender tube was employed to cyclically administer tricaine solution from the petri dish to the oral cavity of zebrafish. The head of the zebrafish was positioned beneath the scanning lens. The imaging process lasted for a duration of less than two minutes. Following the imaging procedure, the zebrafish was transferred to the tank until operculum movement was detected.

The PS-OCT and OCTA imaging results were generated by the methods in our previous studies. The polarization parameters we used in this study included accumulative retardation and degree of polarization uniformity (DOPU) [22]. OCTA data were calculated based on the SSADA algorithm [21].

### 2.4. Organ Segmentation Method

Combined with the intensity and polarization characteristics of each organ in zebrafish, we designed an organ segmentation method, the schematic diagram of which is shown in Figure S3. Firstly, the intensity and DOPU B-scan images are generated. Secondly, the intensity threshold for Air–Skin and Skin–Skull boundaries, the intensity threshold for the Skull–Brain boundary, and the DOPU threshold for Skin–Skull and Skull–Brain boundaries are set. Thirdly, Intensity masks and DOPU masks are generated by corresponding thresholds. Fourthly, DOPU-Intensity masks are generated by multiplying DOPU masks with Intensity masks, respectively. It should be noted that due to some skin pigmentation spots, the segmentation for the Skin–Skull boundary may be affected. Therefore, a morphological opening operation is performed on the DOPU-Intensity mask to reduce the influence of skin pigmentation. Fifthly, the top edge of the Intensity mask is selected as the Air–Skin boundary. The top of the DOPU-Intensity mask is selected as the Skin–Skull boundary. The Bottom of the DOPU-Intensity mask is selected as the Skull–Brain boundary. Finally, these boundaries are smoothed using spline fitting.

Based on the organ segmentation method, we can divide the imaging results into three parts: skin, skull, and brain. The segmentation results are shown in Figure S4.

## 3. Results and Discussion

### 3.1. Pigmentation Imaging for Adult Zebrafish Intra- and Extracranial Regions

Pigmentation is prevalent in all parts of the human body, located in the skin, eye, inner ear, bones, and meninges. Its distribution is related to the regulation of inflammatory processes [23]. Disorders of pigmentation may introduce a variety of diseases, including albinism and vitiligo. In pigment-related studies, zebrafish is a useful model for understanding mechanisms of pattern formation [24]. Currently, investigations into zebrafish pigmentation are primarily focused on its eyes and skin. In this section, we showed intra- and extracranial pigmentation of zebrafish based on the high-resolution multi-functional OCT system.

Our previous research has indicated that the pigmentation exhibits a strong reflection and depolarization due to multiple scattering [19]. The high-resolution multi-functional OCT system utilized in this study can produce the intensity image to represent reflection and the degree of polarization unity (DOPU) image to represent depolarization. The pigmentation was observed to display a high intensity and a low DOPU value [25]. Based on the imaging characteristics of the pigmentation, we generated DOPU-Intensity images to show its distribution. The hue and brightness of DOPU-Intensity images are determined by the DOPU and intensity images, respectively. In DOPU-Intensity images, pigmentation would appear a bright green color.

**Figure 1:**
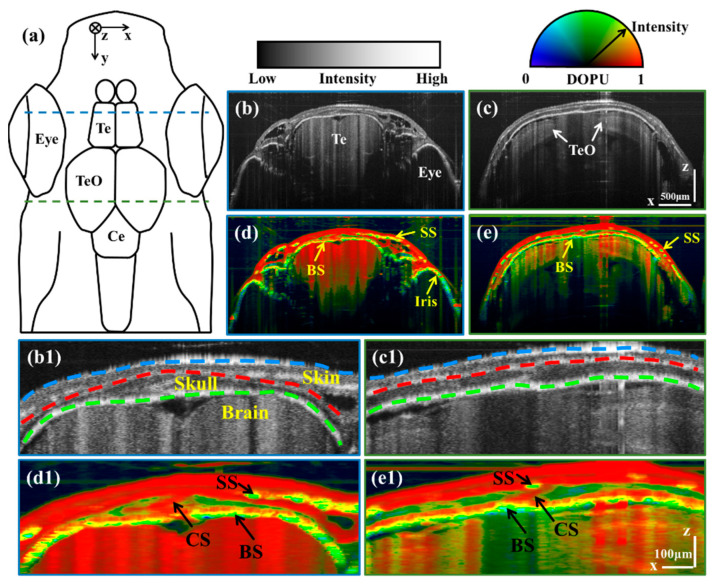
(**a**) is the schematic diagram of the zebrafish. (**b**,**c**) are intensity B-scan images corresponding to Te and TeO regions, respectively. (**d**,**e**) are DOPU-Intensity B-scan images corresponding to Te and TeO regions, respectively. (**b1**–**e1**) are enlarged images corresponding to the cranial regions of (**b**–**e**), respectively. Scale bars of (**b**–**e**) are 500 μm. Scale bars of (**b1**–**e1**) are 100 μm. (BS: Bottom of the skull; CS: Cranial suture; SS: Skin spots; Te: Telencephalon; TeO: Tectum opticum.) (Blue, red, and green dashed lines in (**b1**,**c1**) are Air–Skin, Skin–Skull, and Skull–Brain boundaries, respectively.)

To observe the pigmentation of different organs, we selected B-scan images corresponding to locations marked by blue and green dashed lines in the schematic diagram of the zebrafish [Figure 1a]. Figure 1b,c are intensity B-scan images corresponding to telencephalon (Te) and tectum opticum (TeO) regions, respectively. Figure 1d,e are DOPU-Intensity images corresponding to Te and TeO regions, respectively. It can be seen that pigmentation exists in the skin spots (SS), the bottom of the skull (BS), and the iris. It can be seen that the imaging depth for adult zebrafish head is larger than 500 µm, which has reached the inside of the brain.

Then, we enlarged the pigment-rich cranial region to observe the details. Figure 1(b1–e1) are enlarged images corresponding to the cranial regions of Figure 1b–e, respectively. As shown in Figure 1(b1,c1), three boundaries can distinguish different organs, including Air–Skin, Skin–Skull, and Skull–Brain boundaries (marked with blue, red, and green dashed lines, respectively). The cranial suture (CS) is shown as a slash in the center of the skull, which is identified with black arrows in Figure 1(d1,e1). It can be seen that there are pigmentation deposits in the bottoms of the skin and the skull. The difference is that the pigments in the skin gather and form spots, while the pigments in the skull are evenly distributed at the bottom of the skull. This phenomenon reflects the differences in the degree and form of pigmentation in the skull and skin. Furthermore, although the skull has a low DOPU resulting from its scattering properties, the intensity of the skull is lower than that of the pigmentation due to the transparency of the bone.

The imaging results indicate that a high-resolution multi-functional OCT possesses the potential to serve as a valuable tool for pigmentation-related studies. Currently, research on zebrafish pigmentation primarily concentrates on the skin, with limited attention given to cranial pigmentation. However, for humans, skeletal hyperpigmentation, also known as “black bone”, is considered a rare phenomenon that may be caused by medications and diseases [26]. Due to the small number of reported cases, it is difficult to form a comprehensive understanding of the mechanism of the skeletal hyperpigmentation [27]. Currently, there are studies investigating cranial pigmentation in zebrafish [28]. The utilization of a high-resolution functional OCT system to investigate the formation mechanism of skeletal pigmentation in zebrafish may augment the understanding of the “black bone” in humans.

### 3.2. Tissue-Specific Information in the Skin, Skull, and Brain

**Figure 2:**
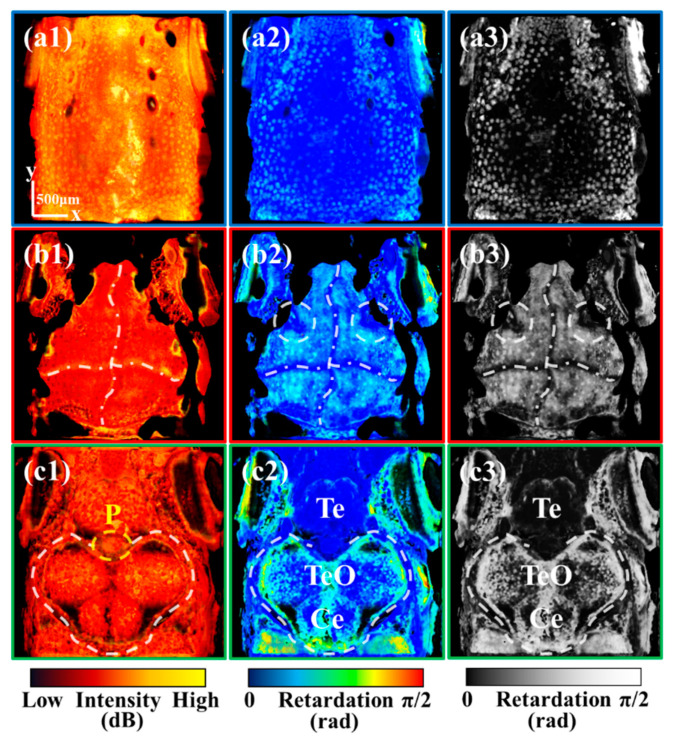
(**a1**,**a2**) are the mean intensity and retardation projections of the skin, respectively. (**a3**) is the image converted from (**a2**) to grayscale. (**b1**–**b3**) are the mean intensity, retardation, and optic axis projection of the skull, respectively. (**c1**–**c3**) are the mean intensity, retardation, and optic axis projection of the brain, respectively. All scale bars are 500 μm. (P: Pineal gland; Te: Telencephalon; TeO: Tectum opticum; Ce: Cerebellum.) (White dashed circles in (**b2**,**b3**) mark cranial sutures at both the left and right sides of the skull. White dash-dotted lines in (**b1**–**b3**) mark cross-shaped cranial sutures.)

One of the extant limitations of conventional OCT is the absence of tissue-specific contrast thereby impeding the identification of distinct tissues. In our prior investigation [19], we found that polarization contrast can help to achieve tissue-specific information. Nonetheless, the physiological structure of zebrafish is intricate and diminutive, making tissue-specific information from different organs potentially confounded. In this section, we used a well-designed organ segmentation method to divide the imaging results into the skin, skull, and brain. Tissue-specific information was extracted from these three parts. Figure 2a–c are the imaging results of the skin, skull, and brain, respectively.

Considering the reflectivity and polarization properties of different tissues, we mainly extracted tissue-specific information through the intensity and retardation images. The retardation image is sensitive to highly birefringent and highly depolarized tissues [19]. Based on these images, we sequentially analyzed what specific information a multi-functional system can present for zebrafish skin, skull, and brain, respectively.

The skin imaging results are presented in Figure 2(a1–a3). Figure 2(a1,a2) are the mean intensity and retardation projections of the skin, respectively. As mentioned in the previous section, the pigmentation spots on the skin exhibit high intensity and high retardation values due to multiple scatterings. Since the low retardation of zebrafish skin, retardation images have high contrast for the pigmentation spots with high retardation. As shown in Figure 2(a3), the retardation grayscale image can generate tissue-specific information about pigmentation spots. The bright spots in the skin are indicative of pigmentation spots. Notably, the pigmentation spots located at the periphery of the zebrafish head are considerably more abundant than those situated on the cranial vertex. This particular distribution of pigmentation spots may be attributed to the migration of pigmented cells, which should be further investigated with our system.

The skull imaging results are presented in Figure 2(b1–b3). Figure 2(b1,b2) are the mean intensity and retardation projection of the skull, respectively. As elucidated in our earlier investigation [19], the cranial suture exhibits a substantial refractive index disparity and minimal birefringence, showing high intensity and low retardation. As shown in Figure 2(b1,b2), the retardation image presents superior contrast for cranial sutures compared to the intensity image. Therefore, the retardation grayscale image [Figure 2(b3)] can serve as the tissue-specific image for cranial sutures. The majority of the dark regions within the cranium correspond to cranial sutures. It is noteworthy that, in addition to the conventional cross-shaped cranial sutures, which are delineated by white dash-dotted lines, there are also cranial sutures at both the left and right sides of the skull, as indicated by two white dashed circles.

Recent research has demonstrated the promising role of cranial sutures in the treatment of human cranial diseases [29]. Investigating the mechanism of cranial suture patency has emerged as a crucial cranial research direction [30]. Studies have shown that all cranial sutures in zebrafish remain patent throughout life [31], and the exploration of the mechanism holds great significance for the study of human cranial sutures. Currently, imaging of the zebrafish cranial sutures is usually based on fluorescence microscopy. Our results demonstrated that a functional OCT can achieve clear and label-free imaging of the cranial suture in vivo. Compared to fluorescence microscopy, functional OCT could greatly streamline zebrafish processing procedures in cranial research.

The brain imaging results are presented in Figure 2(c1–c3), which are the mean intensity, retardation, and retardation grayscale projections of the brain, respectively. As shown in Figure 2(c2,c3), the retardation image can be utilized to discern the region of TeO and cerebellum (Ce) showing high retardation values, which is delineated with a white dashed line. The observed high retardation is attributed to the hyperpigmentation of the upper skull.

It is noteworthy that the cranial sutures marked with two white dashed circles in Figure 2(b3) are located on the left and the right sides of the pineal gland (P) marked with a yellow dashed circle in Figure 2(c1). Some studies have also shown that there is a fixed positional relationship between the pineal gland and the suture of the skull [32]. Given the photoreceptor function of the zebrafish pineal gland, these cranial sutures may have an impact on the light reception of the pineal gland.

### 3.3. Intra- and Extracranial Vascular Imaging

Due to the opaqueness of most adult zebrafish species, current cranial vascular fluorescence imaging methods of adult zebrafish usually require transparent genetically mutated zebrafish (*Casper*) [33], resulting in elevated costs and husbandry challenges. Furthermore, a single type of fluorescent molecule is insufficient for comprehensively labeling the entirety of the intra- and extracranial vessels in adult zebrafish, encompassing both blood and lymphatic vessels. To address this issue, we employed the high-resolution multi-functional OCT to fully depict the intra- and extracranial vessels of opaque wild-type zebrafish.

**Figure 3:**
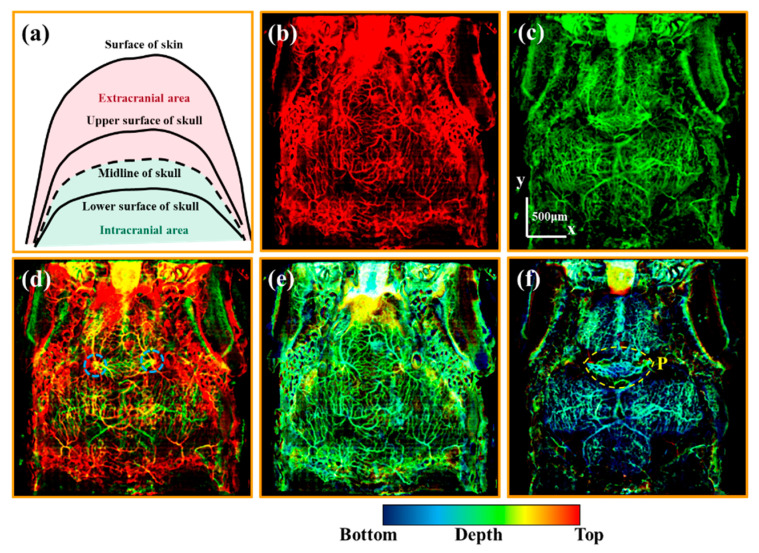
(**a**) is the schematic diagram of the extra- and intracranial regions. (**b**,**c**) are the average OCTA projections of the extra- and intracranial regions, respectively. (**d**) is the merged image of (**b**,**c**). (**e**,**f**) are depth-color encoded OCTA images of the extra- and intracranial regions. All scale bars are 500 μm. (Blue dashed circles in (**d**) mark the vessels in the skull. Yellow dashed circles in (**f**) mark the vessels in the pineal gland.).

To differentiate the intra- and extracranial vessels of the zebrafish, the vascular imaging results were divided into two parts with our organ segmentation method. As shown in the schematic diagram [Figure 3a], the cranial midline marked with a black dashed line is used as a dividing line. The area above the cranial midline is the extracranial area represented in red. The area below the cranial midline is the intracranial area represented in green.

Figure 3b,c are the average OCTA projections of the extra- and intracranial vessels, respectively. The extracranial vascular image [Figure 3b] predominantly displays vessels located in the skin. The intracranial vascular image [Figure 3c] exhibits both meningeal and brain vessels. The merged image of Figure 3b,c reveals a sophisticated vascular network comprising extra- and intracranial vessels in zebrafish, as depicted in Figure 3d. The yellow vessels imply the vessels in the skull, such as the vessels marked with blue dashed circles.

Then, we observed the depth-color encoded OCTA images, whose color indicates the depth information. Figure 3e,f are the depth-color encoded OCTA images corresponding to Figure 3b,c, respectively. As shown in Figure 3e, the extracranial vessels are almost at the same depth at the bottom of the skin. It is noteworthy that Figure 3f shows abundant capillaries in the pineal gland (P) marked with a yellow dashed circle. Additionally, the vessels situated on the left and right sides of the pineal gland are close to the skull and at the same lateral position as the cranial sutures depicted in Figure 2(b3). This phenomenon suggests that the vessels located on the left and right sides of the pineal gland may be linked to the extracranial vessels via the cranial sutures. Subsequently, the following section will concentrate on the observation of these potential channels.

### 3.4. Channels through the Cranial Sutures of Adult Zebrafish

According to the current knowledge, diploic veins are the channels in the diploë between the inner and outer tables of the human skull [34]. Their primary role is to drain the venous blood from the cranial bones to the dural venous sinuses and other intra- and extracranial veins. In cases of venous obstruction, these channels may form important anastomotic connections providing alternative routes for blood flow. To the best of our knowledge, similar channels linking the inside and outside of the skull have not been found in zebrafish. In this section, we confirmed that adult zebrafish possess comparable channels that link the intra- and extracranial vasculatures. Notably, in contrast to humans, the distribution of these channels in zebrafish is primarily concentrated within the cranial sutures.

**Figure 4:**
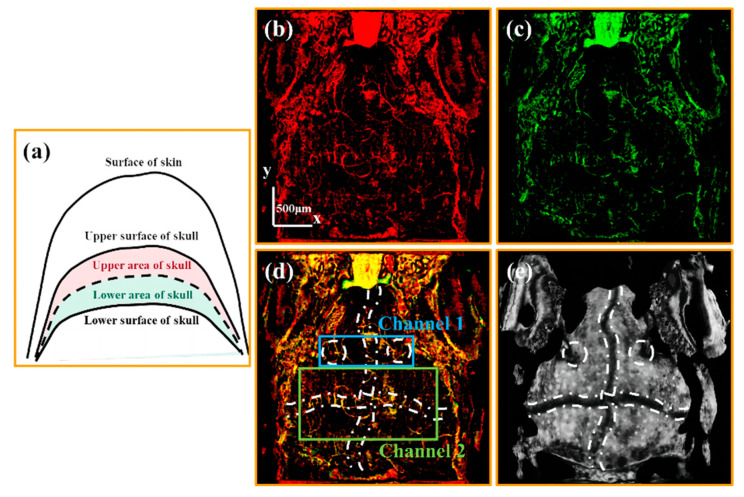
(**a**) is the schematic diagram of the upper and lower area of the skull. (**b**,**c**) are the average OCTA projections of the Upper-Skull layer and the Lower-Skull layer vascular layers, respectively. (**d**) is the merged image of (**b**,**c**). (**e**) is the average retardation projection of the skull. All scale bars are 500 μm. (White dashed circles in (**d**,**e**) mark cranial sutures at both the left and right sides of the skull. White dash-dotted lines in (**d**,**e**) mark cross-shaped cranial sutures.)

To visualize vessels through the skull, it is necessary to obtain imaging results of the cranial region. The upper and lower areas of the skull are defined in a schematic diagram [Figure 4a]. The black dashed line shown in Figure 4a is the cranial midline. We set the area from the cranial midline to the upper surface of the skull as the upper area of the skull, and the area from the cranial midline to the lower surface of the skull as the lower area of the skull. Figure 4b,c are vascular images of the upper and lower areas of the skull, respectively. It can be speculated that the intersection of these two vascular images corresponds to the vessels through the skull.

By merging Figure 4b,c, the channels through the skull are drawn in yellow, as shown in Figure 4d. Comparing Figure 4d with the average retardation grayscale projection of the skull [Figure 4e], we found that these channels mainly exist in the cranial sutures marked with white lines in Figure 4d,e. Based on the distribution of these channels, two distinct types were identified. Channel 1 is observed in the sutures flanking the pineal gland (marked with two white dashed circles), while Channel 2 is found in the cross-shaped sutures (indicated with the white dash-dotted line). Below we further verified these two kinds of channels in detail.

**Figure 5:**
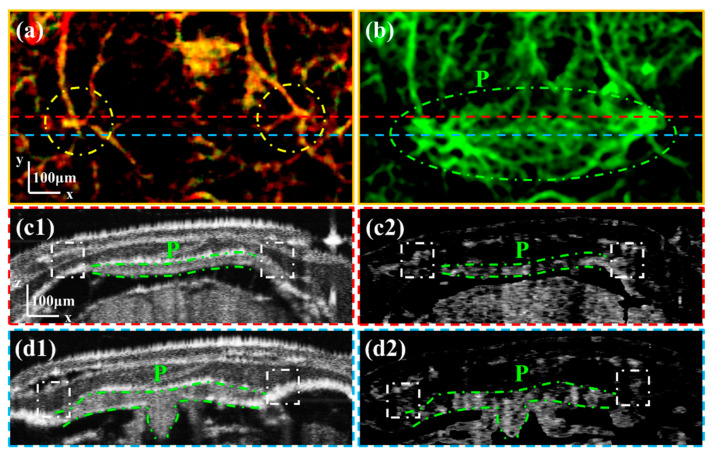
(**a**) is the enlarged image of the area boxed with the blue rectangle in Figure 4d. (**b**) is the enlarged average OCTA image of the Skull–Brain vascular layer. (**c1**,**c2**) are the intensity and OCTA B-scan images corresponding to the position at the red dashed line in (**a**). (**d1**,**d2**) are the intensity and OCTA B-scan images corresponding to the position at the blue dashed line in (**a**). All scale bars are 100 μm. (P: Pineal gland.) (Yellow dashed circles in (**a**) mark the channels within the cranial sutures. White dash-dotted boxes in (**c1**–**d2**) mark the vessels through the cranial sutures.)

Firstly, we observed Channel 1 in the sutures on the left and right sides of the pineal gland. Figure 5a is the enlarged image of the area boxed with the blue rectangle in Figure 4d. The channels through the sutures are boxed with a yellow dash-dotted ellipse in Figure 5a. Figure 5b is the enlarged average OCTA projection of intracranial vessels at the same location as depicted in Figure 5a. The vessels in the pineal gland are circled with a green dash-dotted ellipse in Figure 5b. By comparing Figure 5a,b, it is evident that the channels in cranial sutures are close to vessels on the left and right sides of the pineal gland. To validate these channels, we selected the B-scan images at two locations to observe the cranial vessels in depth.

Figure 5(c1,c2) are the intensity and OCTA B-scan images corresponding to the position at the red dashed line in Figure 5a. Figure 5(d1,d2) are the intensity and OCTA B-scan images corresponding to the position at the blue dashed line in Figure 5a. The area of the pineal gland is circled with green dash-dotted lines. By comparing intensity and OCTA B-scan images, we can see that the vessels on the left and right sides of the pineal gland are through the cranial sutures marked with white dash-dotted boxes. Therefore, it can be confirmed that there are channels on the left and right sides of the pineal gland connecting the intra- and extracranial vasculature.

**Figure 6:**
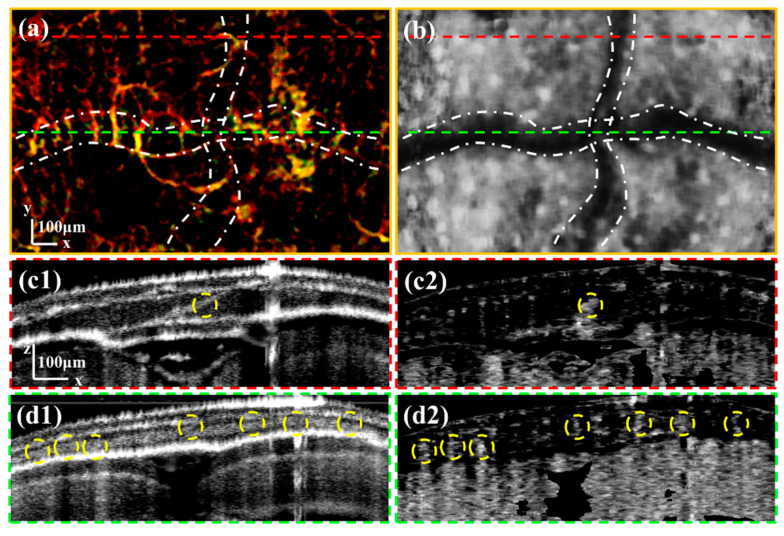
(**a**) is the enlarged image of the area boxed by the green rectangle in Figure 4d. (**b**) is the enlarged average retardation image of the skull. (**c1**,**c2**) are the intensity and OCTA B-scan images corresponding to the position at the red dashed line in (**a**). (**d1**,**d2**) are the intensity and OCTA B-scan images corresponding to the position at the green dashed line in (**a**). All scale bars are 100 μm. (White dash-dotted lines in (**a**,**b**) mark the cross-shaped sutures. Yellow dashed circles in (**c1**–**d2**) mark the channels within the cranial sutures.)

Secondly, we observed Channel 2 in the cross-shaped cranial sutures. Figure 6a is the enlarged image of the area boxed with a green rectangle in Figure 4d. Figure 6b is the average retardation grayscale projection of the skull at the same area shown in Figure 6a. The distribution of channels within the cranial sutures is evident, particularly in the sagittal suture of the cross-shaped cranial sutures marked with white dash-dotted lines. Furthermore, a nearly vertical correlation exists between the orientation of the vessels and the direction of the cranial suture. This phenomenon may be related to the development of cranial sutures.

We selected two positions to verify the channels in the cross-shaped cranial suture. Figure 6(c1,c2) are the intensity and OCTA B-scan images corresponding to the position at the red dashed line in Figure 6a. Figure 6(d1,d2) are the intensity and OCTA B-scan images corresponding to the position at the green dashed line in Figure 6a. We can observe that the vessels in the upper layer do not form artifacts in the vascular imaging of the cranial suture. This is because the thin vessels in the superficial layer have less influence on the backscattered light originating from the deep layer. The distribution of channels within the cranial sutures, marked with yellow dashed circles, exhibits non-uniformity that may be linked to the types of the zebrafish cranial sutures.

Interestingly, the diploic veins of humans do not cross cranial sutures [35], while the channels of zebrafish mainly exist in cranial sutures. This difference may be because humans have diploë between the inner and outer tables of the skull for diploic veins, but the zebrafish does not have a similar structure. Consequently, the presence of channels within cranial sutures in zebrafish may represent an alternative mechanism for establishing connections between the intra- and extracranial vasculatures. Moreover, studies have shown that cranial sutures in zebrafish remain patent throughout the organism’s life [31]. Conversely, the fusion of cranial sutures in humans typically occurs during the third or fourth decade of life. [36]. The channels of zebrafish may play a role in maintaining the patency of the cranial sutures.

In addition, diploic veins drain the venous blood from the cranial bones to the dural venous sinuses [34], which exhibit a close association with meningeal lymphatics. Recent investigations have demonstrated the significance of zebrafish as a crucial model for meningeal lymphatics [12]. The identification of cranial channels in this study may offer novel insights into the mechanism of lymphatic vessel formation.

### 3.5. Monitoring of Traumatic Brain Injury (TBI)

Traumatic brain injury (TBI) is an externally induced injury to the brain that may result in permanent disability or fatality. Because zebrafish maintain a remarkable regenerative ability to repair neural tissue throughout adulthood [37], they have emerged as a valuable model for investigating TBI recovery. However, the impact of TBI on vascular structure and morphology remains inadequately explored [38]. Monitoring the recovery process of a zebrafish’s TBI holds significant research value for investigating the mechanisms of TBI recovery. In this section, the high-resolution multi-functional OCT was utilized to monitor the revascularization process of a penetrating TBI in adult zebrafish.

**Figure 7:**
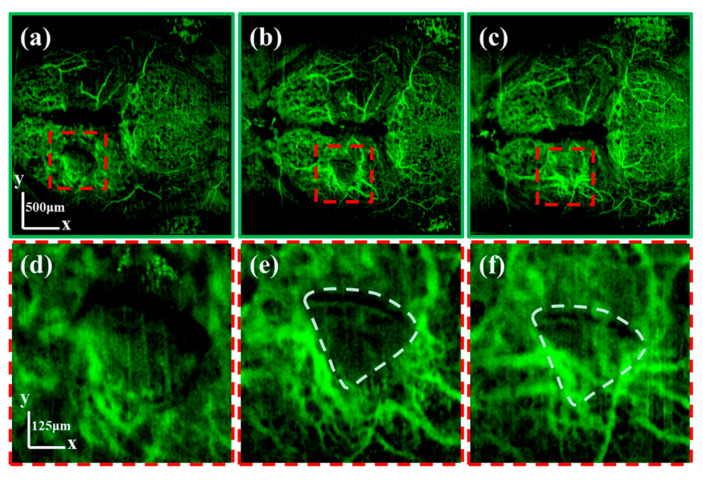
(**a**–**c**) are the average OCTA projections containing the wound location at 0.5, 24, and 48 h after injury, respectively. (**d**–**f**) are the enlarged images of the injured area corresponding to (**a**–**c**), respectively. The scale of (**a**–**c**) is 500 μm. The scale of (**d**–**f**) is 500 μm.

Figure 7 shows the monitoring results of TBI in the zebrafish’s brain (TeO area). The average OCTA projections at 0.5, 24, and 48 h after injury are shown in Figure 7a–c, respectively. Red dashed squares mark the injury area, whose enlarged images are shown in Figure 7d–f, respectively. It can be seen that, 0.5 h after the brain injury, the vessels are in a disorganized state, which is obviously missing in the injury area. However, 24 h after the injury, numerous tiny blood vessels grew and surrounded the injury area, which is marked with a white dashed line in Figure 7e,f. Then, 48 h after the injury, vessels became thicker and crowded into the injury area. Studies have shown that vasodilation is the cause of decreased vascular resistance and increased cerebral blood volume, constituting a factor in vasogenic edema [39]. The thickening of vessels that we observed may be a manifestation of vasodilation. Furthermore, it can be seen that revascularization starts from the outer edge of the brain, which is already rich in blood vessels. This phenomenon may suggest a starting point for revascularization.

The condition of vascular injury is a critical metric for the diagnosis of TBI [40]. Currently, the in vivo assessment of an adult zebrafish’s vascular change after a TBI is mainly accomplished through macroscopic observation of the hematoma, which can only provide qualitative results [41]. Our imaging results showed clear vessel structure and reconstruction process. A high-resolution multi-functional OCT imaging technique can provide high-resolution vascular images to enable the precise assessment and sustained monitoring of the TBI recovery process.

## 4. Conclusions

We employed a high-resolution multi-functional OCT system to conduct intra- and extracranial imaging of adult zebrafish. Through the utilization of this imaging tool, we were able to obtain rich, functional information regarding pigmentation distribution, tissue-specific characteristics, cranial vascular imaging, and TBI monitoring. The results of our investigation revealed many novel physiological structures and pathophysiological processes. Our findings underscore the potential of a high-resolution multi-functional OCT system in facilitating multifaceted intra- and extracranial studies and yielding unique and valuable imaging outcomes.

## Data Availability

The data presented in this study are available on request from the corresponding author. The data are not publicly available due to privacy restrictions.

## Supplementary Materials

The following supporting information can be downloaded at https://www.mdpi.com/article/10.3390/bioengineering10070856/s1: Figure S1: Experimental schematic of the multi-functional OCT system; Figure S2: (a) Experimental schematic of the anesthesia system, (b) photos of anesthesia system; Figure S3: The flow chart of the organic segmentation method; Figure S4: (a1,b1) are B-scan intensity images with the segmentation results in Te and TeO regions, respectively; (a2,b2) are enlarged images corresponding to the orange boxed areas in (a1,b1), respectively; (c1–c3) are three-dimensional rendering images of skin, skull, and brain, respectively.
