# Supplementary material for: Information-Rich Multi-Functional OCT for Adult Zebrafish Intra- and Extracranial Imaging
Source: Bioengineering (Basel). 2023 Jul 19;10(7):856. doi: 10.3390/bioengineering10070856 (PMC10375992; doi:10.3390/bioengineering10070856)
Supplement: Supplementary file 1 [file bioengineering-10-00856-s001.zip › bioengineering-2459558-supplementary.pdf]

Article

Information-Rich Multi-Functional OCT for Adult Zebrafish Intra- and Extra-Cranial Imaging

Di Yang, Weike Wang, Zhuoqun Yuan, and Yanmei Liang *

Institute of Modern Optics, Nankai University, Tianjin Key Laboratory of Micro-scale Optical Information Science and Technology, Tianjin, 300350, China; 9820210118@nankai.edu.cn (D.Y.); wangweike@mail.nankai.edu.cn (W.W.); 1120210110@mail.nankai.edu.cn (Z.Y.)

* ymliang@nankai.edu.cn

Supplementary Materials

1. Construction of the multi-functional OCT system with anesthesia system

The broadband light source (BLM2-D, Superlum) centered at 840 nm has a bandwidth of 100 nm. The light from the source is vertically polarized with a three-paddle polarization controller (PC) and a linear polarizer (POL). Then, the polarized light is separated into the sample and reference arms by a 50:50 non-polarizing beam splitter (BS). In the sample arm, the light passes through a quarter-wave plate (QWP1, at 45°) and a scanning lens (LSM03-BB, Thorlabs) to scan the sample. In the reference arm, the light passes through a quarter-wave plate (QWP2, at 22.5°), a dispersion compensator (DC), and a neutral density filter (ND), and is reflected by a reference mirror (RM). The returned sample light and reference light interfere with each other at the BS. Finally, the interference light is split by a polarizing BS (PBS) into two orthogonal polarization states and acquired by two commercial spectrometers (SP) (Cobra-S 800, Wasatch Photonics).

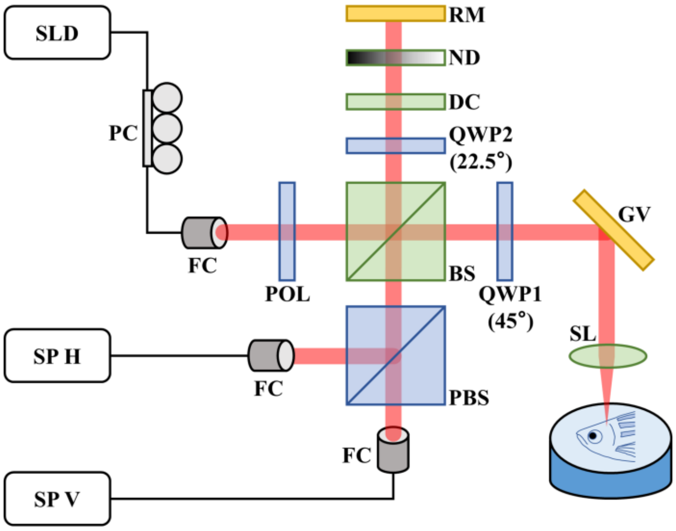

Figure S1. Experimental schematic of the multi-functional OCT system. Polarization optics are drawn in blue. SLD, superluminescent diode; PC, polarization controller; FC, fiber collimator; POL, linear polarizer; BS, non-polarizing beam splitter; QWP, quarter-wave plate; DC, dispersion compensator; ND, neutral density filter; RM, reference mirror; GV, galvanometer scanning mirror; SL, scanning lens; PBS, polarizing beam splitter; SP, spectrometer; H and V, horizontal and vertical polarization state channels, respectively.

2. Construction of the anesthesia system

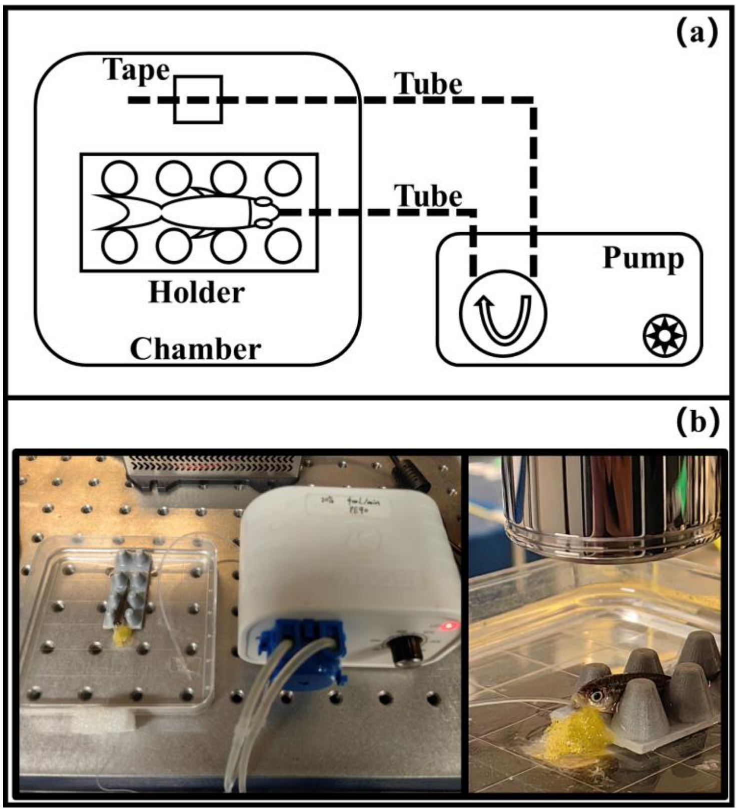

Figure S2. (a) Experimental schematic of the anesthesia system. **(b)** photos of anesthesia system.

3. Organic segmentation method

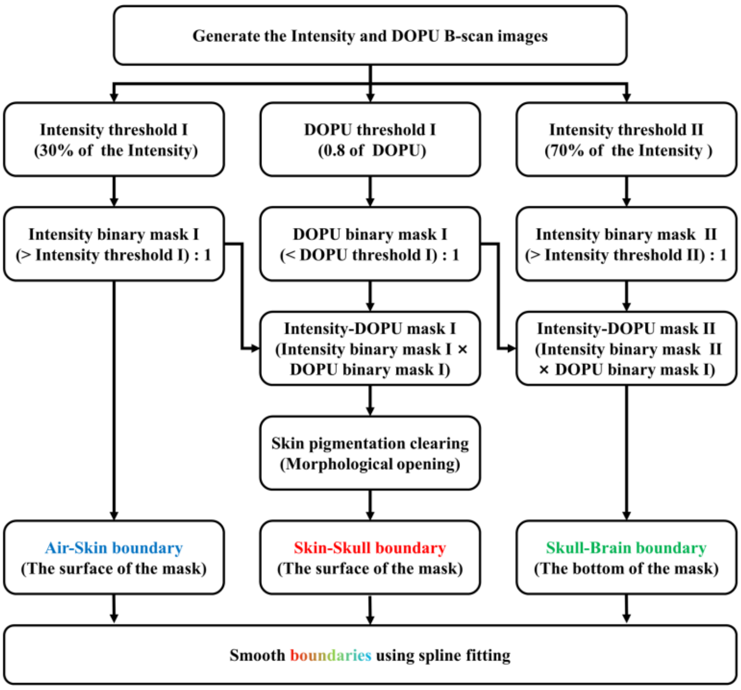

Figure S3. The flow chart of the organic segmentation method

4. Segmentation results of zebrafish head

The segmentation results in Te and TeO regions are shown in intensity B-scan images [Figures S4(a1) and S4(b1)], respectively. Figures S4(a2) and S4 (b2) are enlarged intensity B-scan images, which correspond to areas marked by orange dashed rectangles in Figures S4(a1) and S4(b1), respectively. It can be seen that our organic segmentation algorithm can clearly identify three boundaries.

Based on the segmented boundaries, the zebrafish head can be divided into three layers: skin, skull, and brain. Their three-dimensional rendering images are shown in Figures S4(c1-c3), respectively. Figure S4(c2) shows the shape of the skull and the cross-shaped skull sutures (marked by white dash-dotted lines). Figure S4(c3) shows several areas of the brain including the cerebellum (Ce), tectum opticum (TeO), and telencephalon (Te).

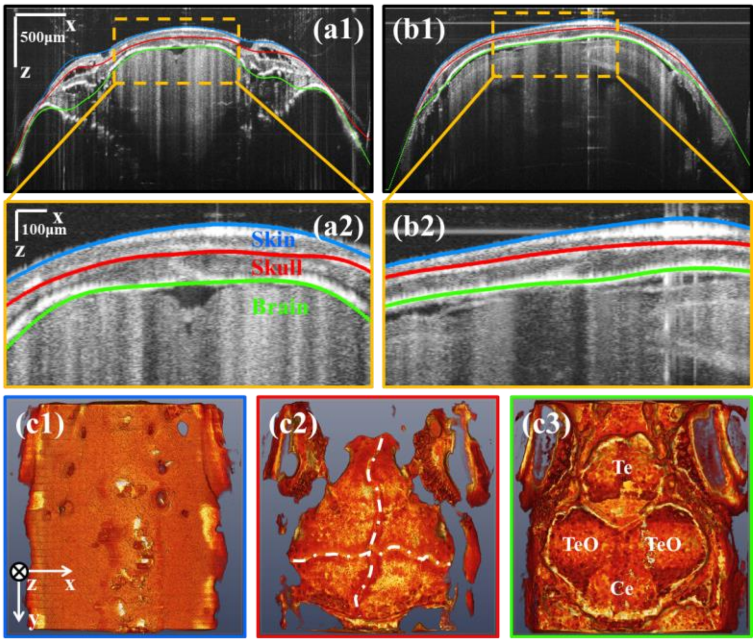

Figure S4. (a1) and **(b1)** are B-scan intensity images with the segmentation results in Te and TeO regions, respectively. **(a2)** and **(b2)** are enlarged images corresponding to the orange boxed areas in **(a1)** and **(b1)**, respectively. **(c1-c3)** are three-dimensional rendering images of skin, skull, and brain, respectively. Scale bars of **(a1)** and **(b1)** are 500 μm. Scale bars of **(a2)** and **(b2)** are 100 μm. (Te: Telencephalon; TeO: Tectum opticum; Ce: Cerebellum)
